# Can Synovial Pathobiology Integrate with Current Clinical and Imaging Prediction Models to Achieve Personalized Health Care in Rheumatoid Arthritis?

**DOI:** 10.3389/fmed.2017.00041

**Published:** 2017-05-03

**Authors:** Frances Claire Humby, Farida Al Balushi, Gloria Lliso, Alberto Cauli, Costantino Pitzalis

**Affiliations:** ^1^Department of Experimental Medicine and Rheumatology, William Harvey Research Institute, Queen Mary University of London, London, UK; ^2^Department of Rheumatology, Royal Hospital, Muscat, Oman; ^3^Dipartimento di Scienze Mediche, Facoltà di Medicina e Chirurgia, Università degli Studi di Cagliari, Cagliari, Italy

**Keywords:** rheumatoid arthritis, synovial membrane, pathology, imaging, personalized medicine

## Abstract

Although great progress has been made in the past decade toward understanding the pathogenesis of rheumatoid arthritis (RA), clinicians remain some distance from a goal of personalized health care. The capacity to diagnose RA early, predict prognosis, and moreover predict response to biologic therapies has been a research focus for many years. How currently available clinical prediction models can facilitate such goals is reviewed in this article. In addition, the role of current imaging techniques in this regard is also discussed. Finally, the authors review the current literature regarding synovial biomarkers and consider whether integration of synovial pathobiology into clinical prediction algorithms may enhance their predictive value.

## Introduction

Rheumatoid arthritis (RA) is characterized by a chronic symmetrical synovitis and underlying erosion of the subchondral bone and cartilage. It is associated with significant morbidity and mortality, with erosive damage directly related to disability and an economic burden for the UK economy alone of around £4 billion. It is now well recognized that the early diagnosis and treatment of RA equates to better long-term outcomes (1). The use of biologic drugs has transformed the treatment of RA, and since infliximab first came into routine clinical practice in the late 1990s, the biologics market for RA has expanded to include more than nine licensed preparations, with significantly more in late stage clinical development. Generally such agents are well tolerated, but adverse events can be severe and fatal and added to this is a high financial cost, with an annual cost of £200 million in the UK alone. Although variable, clinical trial data suggest response rates of around 60% (2) with first biologic treatment, as assessed by the composite clinical assessment tool of the American College of Rheumatology (ACR) 20 response or a fall in disease activity score of 28 joint count (DAS28) of >1.2. However, such level of clinical response is of modest significance to patient functionality, while an ACR 70 response that would enable patients to work and look after their family is reached in only 20–25%. Thus, a large proportion of patients (approximately 80%) are left with no response or significant ongoing disease activity. Additionally, a further proportion of patients will develop secondary failure following an initially promising response, leaving altogether a huge unmet medical need.

In the era of personalized health care, a major focus of rheumatological research has been on predicting prognosis of RA, focusing on three main areas: (i) the early diagnosis and treatment of RA, (ii) predicting prognosis following diagnosis of RA, and (iii) predicting response to biologic therapies. On this basis, a number of clinical prediction models have been developed with variable reports of specificity and sensitivity for both early diagnosis and predicting prognosis of RA.

Synovial tissue is a crucial mediator of cartilage and subchondral bone erosion in RA and as such remains at the epicenter of joint pathology, although its exact place in the hierarchy of disease initiation and/or perpetuation is not entirely clear. In particular, the transition from the pre-clinical phase [breach of tolerance in secondary lymphoid organs (SLOs), mucosal associated lymphoid tissues, e.g., gut (GALT) and bronchial (BALT)] to localizing the disease to the joint is not fully understood. However, once the disease gets hold of the joint, examination of pathobiological specimens from the target organ, similarly to many other branches of medicine, including nephrology, dermatology, gastroenterology, and oncology, may become part of standard clinical care, with management decisions directed by the integration of pathobiology into clinical prediction models. With the advent of new techniques, such as ultrasound-guided synovial biopsy (3), the acquisition of synovial tissue has become a simple and well-tolerated procedure and emulating such a diagnostic/prognostic oncological model a potential goal. It is in this context that this review will discuss the currently available clinical prediction models and address whether the future integration of synovial pathobiology may make personalized health care in RA a more tangible objective.

## Current Clinical Prediction Models

### Predictive Models for the Diagnosis of RA in Early Inflammatory Arthritis

The first clinical prediction model for use in early inflammatory arthritis was that developed by Henk Visser et al. (4). In a study of 524 early arthritis patients, seven variables: (i) symptom duration at first visit, (ii) early morning stiffness (EMS), (iii) arthritis in three or more joints, (iv) bilateral compression pain in the metatarsophalangeal (MTP) joints, (v) rheumatoid factor (RF) positivity, (vi) anti-citrullinated protein antibodies (ACPA) positivity, and (vii) the presence of erosions (hands/feet) were modeled for predictivity and shown to reliably discriminate between self- limiting, persistent non-erosive, and persistent erosive arthritis at 2-year follow-up. A subsequent model, again originating from the Leiden Early Arthritis Clinic (5), was developed for use in patients with early undifferentiated inflammatory arthritis to assess progression to erosive arthritis RA. The authors examined nine clinical variables: (i) sex, (ii) age, (iii) localization of symptoms, (iv) severity of EMS, (v) the tender joint count, (vi) the swollen joint count (SJC), (vii) C-reactive protein (CRP) level, and the presence of (viii) RF, or (ix) ACPA. These were modeled (6) into a 14-point scale in a cohort of 570 early arthritis patients. They demonstrated that the positive predictive value for the development of RA with a score of 8 or greater was 91% and that the negative predictive value for patients with a score of 6 or lower was 84% and, further, that virtually none of the patients with a score of 3 or less were ultimately diagnosed with RA. This prediction rule has been validated in a number of other patient cohorts (7, 8) with similarly encouraging results. It should be noted, however, that 25% of patients in the study had a score between 6 and 8 and for these patients the chance of developing RA or not was equal, indicating the need for improvement of available predictive models in a quarter of patients.

The recognition that the 1987 ACR classification criteria for RA were unable to reliably diagnose patients with early disease led to the development of the ACR/EULAR 2010 classification criteria for RA (9), a process that incorporated a data-driven approach with consensus opinion. The 2010 classification criteria weight the acute phase response [erythrocyte sedimentation rate (ESR)/CRP], joint involvement (number and size affected), duration of symptoms (>6 weeks), and seropositivity for RF/ACPA antibodies. These criteria (along with the van der Helm and Visser criteria) have most recently been explored in the REACH study cohort (10) with outcome measures defined as methotrexate use and persistent disease at 12 months. When comparing the ACR/EULAR 2010 criteria with the van der Helm and Visser scores, a superior sensitivity (0.74 vs 0.1 vs 0.59), although lower specificity (0.66 vs 1.0 vs 0.93) was demonstrated for the ACR/EULAR criteria (10). Currently, the individual criteria of the ACR/EULAR 2010 criteria are being analyzed within other early arthritis cohorts with the aim of further improving its diagnostic value (11).

### Predictive Models for the Prognosis of RA

Once RA is diagnosed, predicting the subsequent clinical course remains a challenge and in particular those patients at risk of rapid radiographic progression. Although a number of factors including seropositivity for RF/ACPA, baseline radiographic erosions, and high inflammatory markers associate with a higher erosive load (12–16), none are sufficient independent predictors of outcome (15). A number of clinical prediction models have, therefore, been developed in an attempt to overcome this difficulty (14, 17, 18) with data in general demonstrating that combining factors improves predictive power. For example, a model combining ACPA, sex, ESR, and IgM RF, was reported to have an accuracy of 73.6% to predict radiographic progression (19). A more recent report has suggested that integrating easily accessible clinical and laboratory variables (28 SJC, RF, and CRP or ESR) into a visual matrix model enables identification of those patients at risk of rapid radiological progression (6). The same visual matrix model was subsequently validated in an early arthritis cohort by Durnez et al. (20) to examine whether its application would improve therapeutic decision-making, but although it appeared to prevent overtreatment in the population studied, the predictive capacity for rapid radiological progression was low. Finally, this model (6) was also examined alongside two other prediction models, the BEST (21) and the SWEFOT model (19) in an observational RA cohort (22), and all three were found to have limited power to predict rapid radiological progression. More recently, a simplified model incorporating three clinical parameters has been reported although sensitivity/specificity of prediction of radiographic damage was less with an AUC of 0.75 (23).

Finally, although not specifically a clinical model the widely validated multi-biomarker disease activity score, incorporating 12 serum biomarkers has recently been demonstrated to reliably predict radiographic progression at 12-month follow-up in a cohort of early RA patients although requires validation in further cohorts (24).

## Incorporating Imaging into Clinical Prediction Models for the Diagnosis and Prognosis of RA

Magnetic resonance imaging (MRI) and ultrasonography (US) have shown utility both in the early diagnosis of inflammatory arthritis and in monitoring its progression, and as such, a number of studies have examined their use as diagnostic/prognostic markers. MRI has been demonstrated to identify bone erosions early (25) and, furthermore, as a sensitive means to detect synovitis and bone marrow edema (26, 27). What is currently unclear, however, is how MRI functions as a tool to diagnose RA or further to predict its prognosis. Indeed a recent systematic review by Suter et al. (28) found no consensus on early MRI diagnostic criteria for RA and further concluded that bone marrow edema was the only significant predictor of radiographic progression (29). This is line with results from Duer-Jensen et al. (30) who examined predictive MRI features in an early undifferentiated arthritis cohort and identified BME as an independent predictor of subsequent RA development. The authors went on to integrate BME into a clinical matrix prediction model and directly compared the performance with the previously described van der Helm-van Mihl model (vdHvM model) (5) and reported an enhanced performance in this cohort with the former model correctly identifying the development of RA in 82 vs 60.2% of patients. The importance of BME was also highlighted by Tamai et al., who demonstrated in an early arthritis cohort that the combination of BME and ACPA positivity was equivalent to the application of the vdHvM model (31). Furthermore, the demonstration in a RF-negative cohort of 40 early arthritis patients (32) that MRI features of synovitis, erosions, and BME were significantly more sensitive than ACPA in correctly identifying RA patients suggest a potentially valuable role for MRI in this particular cohort.

Magnetic resonance imaging continues to be an expensive imaging modality, however, and is also time consuming and cumbersome for the patient. Conversely US can be performed swiftly and relatively cheaply within outpatient clinics, a considerable advantage, particularly as immediate treatment decisions can then be made following the scan mostly carried out and interpreted by rheumatologists. Again, it has been shown to be a sensitive tool to identify early erosions and synovitis (33). An initial report by Freeston et al. demonstrated a diagnostic benefit to adding ultrasonographic examination of the metacarpalphalangeal (MCP) joints, wrists, and flexor tendons to conventional clinical tools (RF/ACPA status, ESR/CRP, radiographic damage) for the diagnosis of very early inflammatory arthritis (34). The predictive algorithm appeared particularly helpful in seronegative patients, with a probability of certain diagnosis increasing from 30 to 94% once ultrasonographic features were incorporated. Interestingly, a recent study performed in very early arthritis incorporated US (of the MCP, wrist, and MTP joints) into both the van der Helm clinical prediction model and the ACR/EULAR 2010 classification criteria and demonstrated that incorporating ultrasound increased sensitivity for both clinical prediction models (35). Conversely, however, data from a real-life early arthritis clinic (36) cohort of 379 patients did not demonstrate a benefit in the addition of US into a risk metric derived from 12 clinical and serological parameters for the prediction of the development of either persistent inflammatory arthritis or RA.

### Clinical Predictive Models for Response to Biologic Treatment in RA

As already mentioned predicting the response to biologic agents is critical to the management of patients in order to optimize response and limit serious side effects. Although there are no clearly validated models for any of the biologic agents, a number of biomarkers have been explored. First, seropositivity for RF and ACPA and the primary response to anti-TNF agents has been examined in a number of cohorts (37–39) with mixed results, and thus at present, neither can be recommended as reliable biomarkers. Interestingly, however, it is becoming increasingly clear that seropositivity for RF and/or ACPA does enhance response to rituximab (40–42). Similarly, enhanced retention to abatacept in patients seropositive for RF and/or ACPA has also been observed in two independent cohorts (43, 44). Despite these observations, significant clinical challenges remain in predicting response to rituximab or abatacept with wide variability seen in response rates within the seropositive groups. Finally, preliminary data from an observational cohort of 530 RA patients have suggested that high levels of CRP retention with tocilizumab (45).

## What Can Synovial Pathobiology Add to Current Clinical Prediction Models?

Thus, while clinical prediction models offer potential (summarized in Table 1), they have a number of limitations, and at present, none are used in routine clinical practice, primarily because of lack of validation in large routine patient cohorts outside specialized centers and levels of sensitivity/specificity. These are important considerations and with a long-term goal of personalized health care much effort has gone into the search for additional biomarkers.

**Table 1:** **Summary of diagnostic clinical prediction models in rheumatoid arthritis**.

Clinical prediction model	Variables	Target population	Outcome	Sensitivity/specificity	Validated on external cohorts yes/no
Visser et al. (4)	i. Symptom duration, ii. early morning stiffness (EMS), iii. arthritis in >3 joints, iv. +ve metatarsophalangeal compression test, v. RF+ve, vi. ACPA+ve, vii. presence of erosions	Inflammatory arthritis	Self limiting vs persistent non-erosive (PNE), persistent erosive (PE) at 2-year follow-up	AUC 0.84 SI vs PE	Yes, e.g., Ref. (10)
				AUC 0.91 PNE vs PE (4)	
Van der Helm-van Mil et al. (5)	i. Sex, ii. age, iii. localization of symptoms, iv. severity of EMS, v. tender joint count, vi. swollen joint count, vii. C-reactive protein, viii. RF+/− or ix. ACPA+/−	Undifferentiated arthritis	Erosive disease	AUC 0.87 (5)	Yes, e.g., Ref. (7, 10)
ACR/EULAR 2010 criteria, Aletaha et al. (9)	i. ESR/CRP, ii. joint involvement (number and size affected), iii. duration of symptoms (>6 weeks), iv. RF/ACPA+ve	Inflammatory arthritis	Persistent arthritis	Sensitivity 0.71	Yes, e.g., Ref. (10, 46)
				Specificity 0.65 (11)	

*Variables, target population, outcome assessed, and sensitivity and specificity of each model are described*.

**Figure 1:**
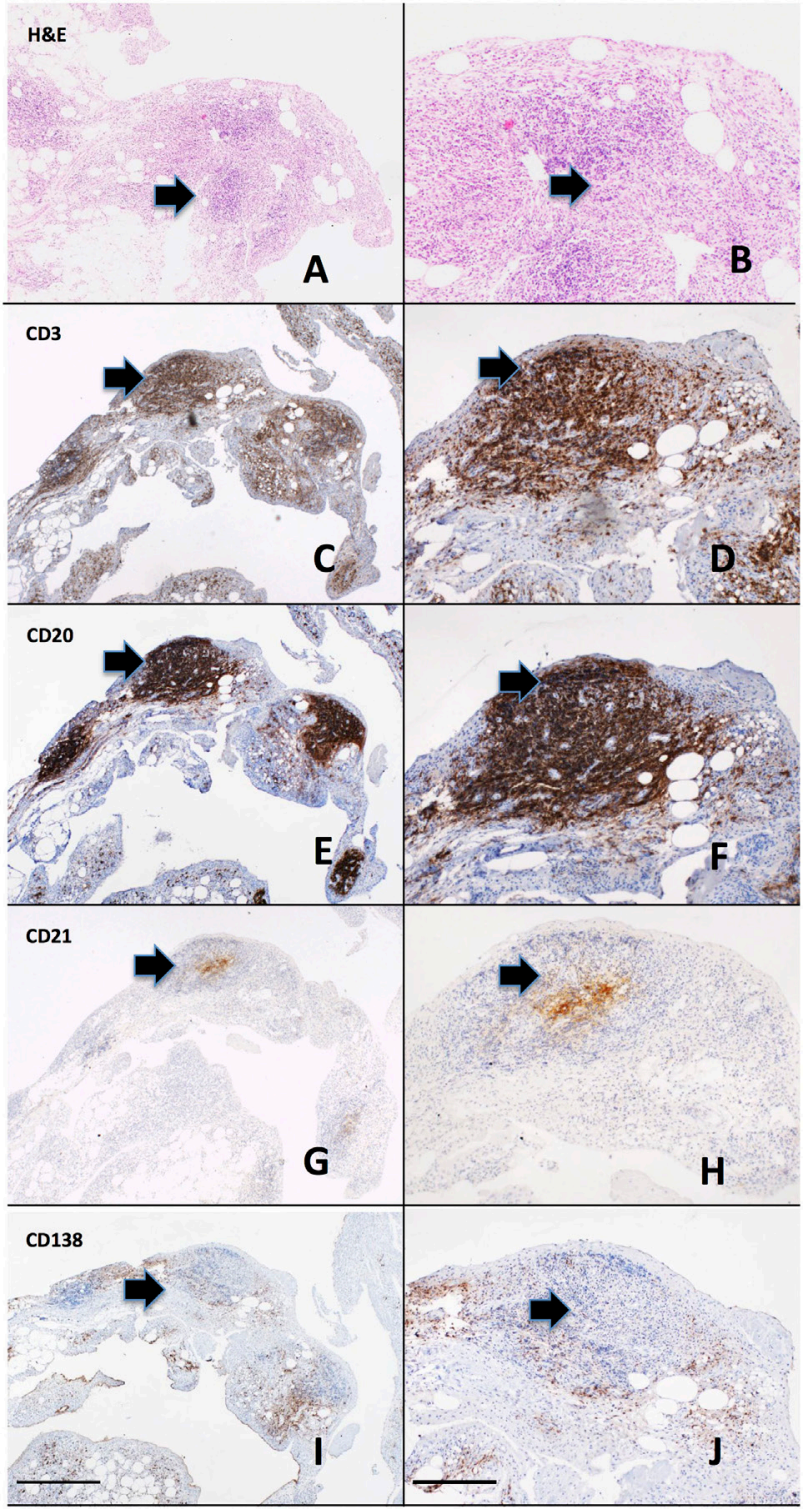
**Ectopic lymphoneogenesis within the rheumatoid synovial membrane**. The synovial inflammatory cell infiltrate has been demonstrated to organize into discrete organized clusters clearly visible following routine hematoxylin and eosin staining **(A,B)**. The aggregate is primarily composed of T cells [CD3, **(C,D)**] and B cells [CD20, **(E,F)**] with a central follicular dendritic cell network [CD21, **(G,H)**]. Plasma cells [CD138, **(I,J)**] are shown surrounding the aggregate. Sequentially cut 3 µm sections of paraffin embedded rheumatoid arthritis (RA) synovial tissue from a patient with early RA are shown. Arrows indicate regions of positively stained cells. Left hand panel 4× magnification, right hand panel 20×, scale bar 200 µm.

Rheumatoid arthritis is characterized by a thickened synovial membrane infiltrated by a diverse array of inflammatory cells, including macrophages, T and B lymphocytes, and NK cells associated with the proliferation of resident synovial fibroblasts. One of the characteristics of rheumatoid synovial tissue is the frequent capacity of the inflammatory cell infiltrate to organize into aggregates, a process termed ectopic lymphoneogenesis (Figure 1). These aggregates are primarily composed of B and T lymphocytes, macrophages, and frequently follicular dendritic cells (FDCs). Such organized ectopic lymphoid structures (ELS) within the target organ of an autoimmune disease are not restricted to RA and indeed are seen within the salivary glands of patients with Sjogren’s syndrome (47), the meninges of patients with multiple sclerosis (48) and in the thyroid of patients with autoimmune thyroid disease (49). Within rheumatoid synovial tissue, ELS can appear as tightly organized clusters closely resembling SLOs, expressing high endothelial venules and CD21+ follicular dendritic cells (50) (Figure 1). Following these observations, much debate has focused on their pathophysiological significance. Indeed, a direct functional role in inflammation and autoantibody production for synovial ELS in RA pathogenesis has come from a number of studies including our own work using the Human RA SCID mouse model (51). We demonstrated that synovial graft ELS support the proliferation and differentiation of B cells as well as the expression of activation-induced cytidine deaminase, an enzyme critical for the processes of class switch recombination and affinity maturation of antibodies (52), which correlated with human ACPA titers (IgG) within mouse serum. In addition, grafts characterized by the presence of ELS (vs ELS−ve grafts) showed higher level of production of B cell growth factors, TNF alpha, and RANK-ligand, supporting a direct functional role for autoantibody and pro-inflammatory mediators production by such structures. Of considerable importance, it should be noted that these events occurred in the absence of new immune cells infiltrating the grafts (SCID are immune deficient), indicating that ELS contribute to disease pathogenesis *via* protracted self-sustained immune activation. Further evidence includes the expression of RANK-ligand by activated T cells within synovial aggregates (53).

Direct evidence for a role in pathogenesis has also been suggested from clinical studies: a significant association was reported in established RA between synovial aggregates and erosive burden (54), and in a prospective cohort of early arthritis, increased synovial B cells were associated with the subsequent development of RA (55). However, the same authors (56) and others in larger cross sectional cohorts have found no association between synovial aggregates and radiological damage (57) and a recent prospective study of early arthritis patients could not confirm either a diagnostic or prognostic role for aggregate synovial pathotypes (58). This has led a number of authors to conclude that lymphoid aggregates are a byproduct of inflammation, rather than a key component of the pathological process (59). However, interpretation of these data is complicated by a number of issues. First, no specific criteria are consistently used between studies to define aggregate synovial tissue histologically and as such the definition of ELS has been variously used to refer to aggregates of differing size and/or expressing different specific immunological cells (e.g., FDC, T cells, B cells). Further, comparing data from cross sectional studies including patients with varying (a) disease duration, (b) radiographic damage, and (c) treatment exposure (different therapeutic agents may influence synovial pathobiology differentially) has inherent methodological discrepancies. Finally, excessive prevalence of synovial tissue from the knee, as is the case with a number of studies, may introduce systematic bias, by including those patients with the most aggressive disease (60). These are crucial considerations, since a significant amount of data suggest that the induction of specific pathways related to lymphoid organization within the synovium (51, 53, 61, 62) may have short- and/or long-term effects on disease evolution. Thus, this concept requires further examination in a large-scale prospective study of early arthritis patients naïve to all therapy.

## Synovial Pathotype as a Predictor of Response to Therapy

Clearly, given the relative ease of access, the identification of biomarkers within peripheral blood would be preferable to those from synovial tissue. However, the search for peripheral biomarkers for response to TNF inhibitors (TNFi) has so far been challenging (63–67), and overall, it can be concluded that at present the currently examined peripheral blood biomarkers lack either sufficient sensitivity or specificity for predicting response to TNFis. Notably, however, the type 1 interferon signature has been identified as a potential marker of resistance to rituximab therapy in RA (68), but this still requires further validation in independent cohorts. The search for synovial biomarkers has, therefore, been driven by the ongoing challenge of selecting the correct biologic in routine clinical practice, on the background of a burgeoning market for biologics usage in RA and by the limited utility so far demonstrated by peripheral blood biomarkers or clinical parameters.

**Figure 2:**
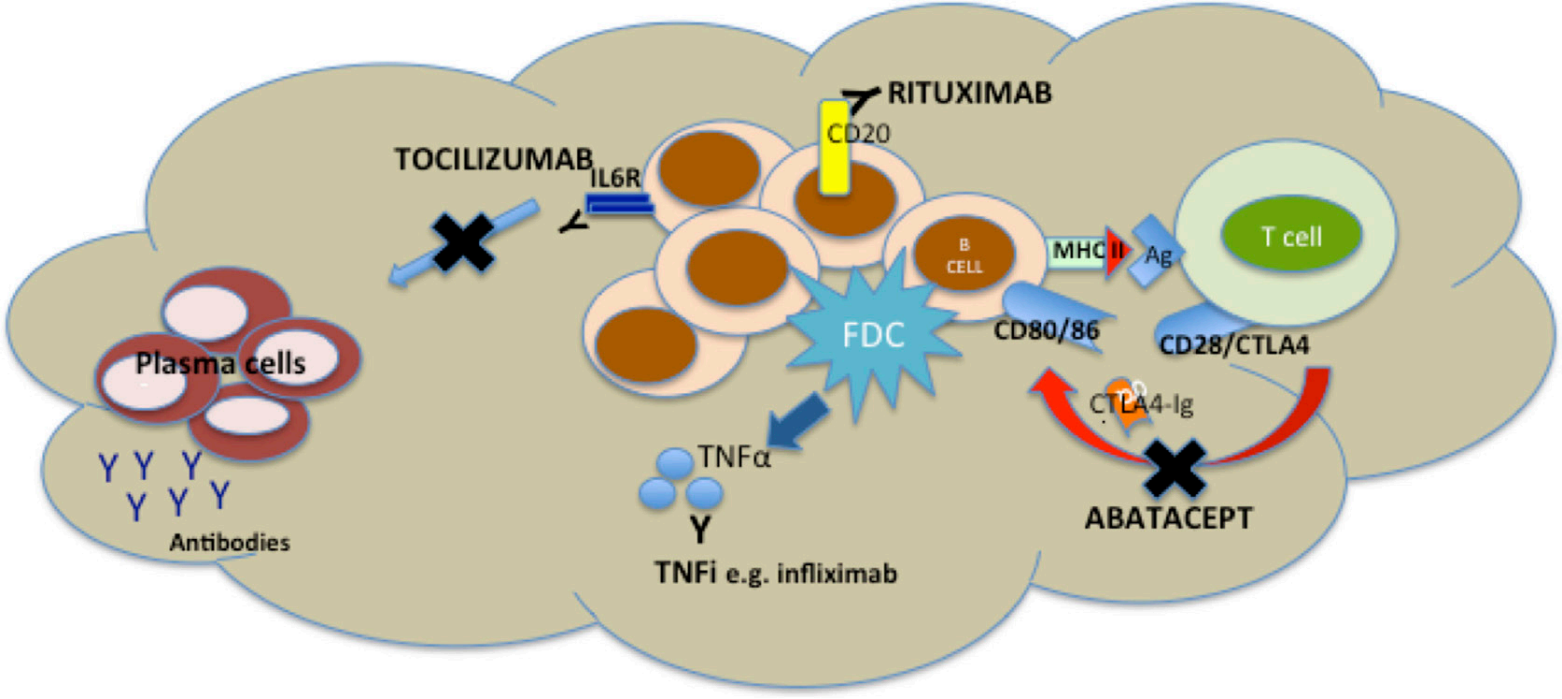
**Mechanisms of disruption of ectopic lymphoneogenesis within rheumatoid synovial tissue *via* currently licensed biologic agents for rheumatoid arthritis (RA)**. Disruption of ectopic lymphoneogenesis within the rheumatoid synovial membrane may be mediated by a number of biologic agents licensed for the treatment of RA and, therefore, has a putative role as a biomarker of response/resistance to biologic therapies. Rituximab induces death of CD20+ B cells *via* Ab or cell-mediated cytotoxicity, phagocytosis, or cell lysis. Abatacept, a CTLA-Ig fusion protein, prevents endogenous CTLA from binding to CD80/86 on B cells (functioning as antigen presenting cells) and so prevents co-stimulatory signals to the B cell. TNF inhibitors (TNFis) (inhibitors) such as infliximab bind to soluble and membrane-bound TNFα released by follicular dendritic cells (FDCs), and so inhibiting FDC-mediated B cell attraction. Tocilizumab inhibits binding of IL6 to its receptor, preventing IL6-mediated B cell proliferation and differentiation.

TNF inhibitors are the most widely used biologic agents and have been in routine use for the longest and as such synovial pathobiological changes are probably the most exhaustively studied. TNFα+/− LTα are inhibited by TNFi drugs, and both cytokines are key mediators of both the induction and maintenance of SLOs (69), so the capacity for modulation of synovial aggregates by TNFi has the potential to dissect the pathophysiology of the disease (Figure 2) as well as identification of biomarkers. Results from two groups have produced somewhat opposite results. An initial report from Cañete et al. (57) investigated the relationship between response to TNFi and the identification of large B cell aggregates with a pretreatment arthroscopic synovial biopsy in 86 patients. Further, in a subgroup of 24 patients, the capacity of TNFi to modulate B cell aggregates and the relationship with clinical response was explored. The data suggested that synovial lymphoneogenesis was an independent negative predictor of response to therapy, and response to treatment was associated with regression of ELS. However, a subsequent report from Klaasen et al. (70) in which synovial biopsies were performed on 97 patients prior to commencing treatment with infliximab identified the presence of lymphocyte aggregates as positive predictors of response at 16 weeks. There are a number of reasons for such discrepancies between the two groups including patient cohorts differing in terms of past TNFi exposure, disease duration, and variable follow-up times. In addition, a different definition of aggregate positive synovial tissue was used within each study, and the uniform use of a single TNFi was not consistent either within (57) or between the studies, a crucial consideration as increasing data suggest that efficacy of TNFis may involve more complex mechanisms than the simple division of drugs into either TNF antibody or TNF receptor (71). Interestingly, however, when the presence of synovial lymphocytic aggregates was added into a clinical prediction model (70) with TNF expression within the synovium, baseline DAS 28 score, and ACPA positivity, the power to predict response increased from 19 to 29%. A further potential synovial biomarker was identified when a preliminary study identified pretreatment levels of synovial TNF as a positive predictor of response to treatment (72) and a subsequent synovial-based study of 143 patients (73) supported these results with TNF expression within the synovial sublining explaining about 10% of the variance in response to therapy when multivariate linear regression was used to investigate synovial markers. Further, when a clinical prediction model incorporating both disease activity and TNF expression with the synovial sublining was formulated, 17% of variance in response to therapy could be explained (73). Thus, although at present, we remain some distance from personalized health care, these studies demonstrate proof of concept that in the long term the integration of synovial pathobiology into clinical prediction models may prove to be a useful clinical tool.

Whole tissue analysis using microarray technology has also been investigated to identify markers of response to therapy with such technology having the advantage of providing multiple simultaneous biomarkers. Its use was supported by two studies: the first where synovial tissue available from 10 patients pre- and post-TNF therapy (74) was examined and suggested a differential expression of genes between responders and non-responders and modulation of transcription profiling following treatment. The second study including 18 patients again suggested a differential expression of predominantly inflammatory genes in TNFi responders (75). Conversely, another group reported in a biopsy-based study of 25 patients that a gene signature characterized predominantly by markers of cell division and immune activation predicted response to therapy (76). A large study of synovial tissue from 62 RA patients was established to attempt to identify further biomarkers of response using transcriptional analysis (77). This study did identify 38 genes associated with good response, but only within aggregate positive patients. Interestingly, more recently published data from Dennis et al. (78) have utilized whole tissue microarray as a tool to characterize the molecular profile of three previously reported (79) pathobiological synovial subgroups: myeloid, fibroid, and lymphoid subtypes. The same authors (78) also went on to identify two soluble serum markers associated with synovial pathotypes: ICAM1 (myeloid) and CXCL13 (lymphoid), and demonstrated that each was found to predict response to adalimumab (ICAM1) and tocilizumab (CXCL13). Such data require validation in larger prospective cohorts but highlight that disease heterogeneity may be more complex than that dissected by simple histopathological assessment of synovial tissue and, furthermore, suggest the potential role for synovial tissue in rationalizing choice of peripheral biomarkers. Therefore, it seems likely that any robust prediction model will have to incorporate multiple pathobiological as well as clinical biomarkers.

Thus, at present, despite these observations, the capacity to predict response to TNFi remains remote to practicing clinicians. What seems crucial to stress, particularly with an ever-increasing number of TNFis available, each with unique pharmacological characteristics and capable of modulating unique pathobiological processes, is that extrapolating biomarkers from individual TNFis to the whole group will likely prove a futile exercise. What is needed are large-scale pathobiological studies conducted in patients naïve to all biologic therapy and focusing on individual TNFis, this approach promises a unique opportunity to dissect disease mechanism in parallel with identifying novel biomarkers.

Rituximab, a monoclonal antibody against CD20, has demonstrated efficacy in RA, with response rates of around 60% (80). Although seropositivity for RF and/or ACPA has emerged as a positive predictor of response to treatment following a subgroup analysis of a number of studies (81–83), outcome to treatment remains unpredictable. Clinical studies pre and post rituximab therapy have demonstrated a number of potential pathways within synovial tissue that may explain the heterogenous clinical response seen despite apparent complete depletion in the peripheral blood. First, in a small study of 13 patients with synovial biopsies taken prior and at 8 weeks following standard rituximab treatment a trend toward clinical response and depletion of both synovial B cells and immunoglobulin synthesis was demonstrated (84). Second, a study of 24 patients identified reduction in the number of plasma cells, potentially originating locally within the synovial tissue, 16 weeks post therapy as a predictor of response to therapy (85). Finally, the identification of circulating pre plasma cells, by high sensitivity FACs analysis following the first cycle of rituximab associated with a poor response to treatment (86). Such cells are thought to originate from solid tissue, e.g., bone marrow, though the contribution of autoreactive clones escaping from the synovium may also play a role. It may well be concluded from these studies that modulating the functional B cell activity within synovial tissue has an important contribution on response to treatment, a hypothesis that is currently being explored by the authors of this paper through two randomized biopsy driven clinical trials (a) the National Institute for Health Research: *R*esponse, *R*elapse, *R*esistance to *R*ituximab (R_4_RA) study and (b) *S*tratification of Biologic *T*herapies for *R*heumatoid *A*rthritis by *P*athobiology (STRAP) study jointly funded by the Medical Research Council and Arthritis Research UK.

Early data on the synovial response to abatacept, a specific modulator of T-cell activation *via* inhibition of the CD80/86-CD28 interaction, have suggested a characteristic response in patients responding to treatment. In the study involving 16 RA patients with synovial tissue obtained prior and 16 weeks following the initiation of therapy, a significant downregulation of a number of pro inflammatory genes and a specific reduction in synovial CD20+ B cells was seen in patients responding to treatment (87). Further studies are awaited.

## Conclusion

A number of major advances in the management of RA have been made in the past decade. Importantly, overwhelming evidence now supports early intervention (1). The introduction of biologic drugs into the therapeutic armamentarium has been associated with a paradigm shift in treatment goals, from an often previously ill-defined response to therapy to disease remission or even cure. Such aims require early diagnosis and to target biologic treatments appropriately, to patients with the worst prognosis and to those most likely to respond to individual agents. The currently available clinical prediction models are insufficiently sensitive or specific to allow reliable early diagnosis or indeed predict those with the worst prognosis and so far biomarkers for response to biologics are widely lacking. Although the simple pathological characterization of synovial tissue into subtypes seems unlikely to aid prognosis *per se*, an enormous number of questions remain before fully understanding how the heterogeneous synovial pathotypes integrate with disease pathogenesis and ultimately the heterogeneity of clinical phenotypes of disease. The complexity and diversity of RA pathogenesis is perhaps as such that would require more integrated and modular approaches as needed for cancer medicine (79, 88, 89). What is clear from the current conflicting data is that future synovial biopsy studies need to be conducted on large cohorts, including early arthritis patients naïve to DMARD therapeutic intervention. Significant progress in minimally invasive ultrasound-guided techniques which are easily carried out by rheumatologists, safe and well tolerated by patients, and allow for the acquisition of synovial tissue both from small and large joint in most patients will facilitate the conduction of such studies. These may not only predict prognosis but have the potential to predict response to future therapeutic intervention at first presentation. It seems realistic to suggest that in future synovial pathobiology will be integrated into current clinical prediction models with positive benefits for patient care and health economics through accurate patient stratification.

## Author Contributions

All authors substantially contributed to the conception/design, acquisition, and analysis of the work. All authors contributed to drafting of the work and gave final approval of the version to be published and are all in agreement to be accountable for all aspects of the work in ensuring that questions related to the accuracy or integrity of any part of the work are appropriately investigated and resolved.

## Conflict of Interest Statement

The authors declare that the research was conducted in the absence of any commercial or financial relationships that could be construed as a potential conflict of interest.

## References

[1] Breedveld FC Combe B. Understanding emerging treatment paradigms in rheumatoid arthritis. Arthritis Res Ther (2011) 13(Suppl 1):S3. 10.1186/1478-6354-13-S1-S3 21624182 PMC3123964

[2] Bergman GJ Hochberg MC Boers M Wintfeld N Kielhorn A Jansen JP. Indirect comparison of tocilizumab and other biologic agents in patients with rheumatoid arthritis and inadequate response to disease-modifying antirheumatic drugs. Semin Arthritis Rheum (2010) 39(6):425–41. 10.1016/j.semarthrit.2009.12.002 20223500

[3] Kelly S Humby F Filer A Ng N Di Cicco M Hands RE Ultrasound-guided synovial biopsy: a safe, well-tolerated and reliable technique for obtaining high-quality synovial tissue from both large and small joints in early arthritis patients. Ann Rheum Dis (2013) 74(3):611–7. 10.1136/annrheumdis-2013-204603 24336336

[4] Visser H Le Cessie S Vos K Breedveld FC Hazes JM. How to diagnose rheumatoid arthritis early: a prediction model for persistent (erosive) arthritis. Arthritis Rheum (2002) 46(2):357–65. 10.1002/art.10117 11840437

[5] van der Helm-van Mil AH Cessie SL Van Dongen H Breedveld FC Toes RE Huizinga TW. A prediction rule for disease outcome in patients with recent-onset undifferentiated arthritis: how to guide individual treatment decisions. Arthritis Rheum (2007) 56(2):433–40. 10.1002/art.22380 17265478

[6] Vastesaeger N Xu S Aletaha D St Clair EW Smolen JS. A pilot risk model for the prediction of rapid radiographic progression in rheumatoid arthritis. Rheumatology (Oxford) (2009) 48(9):1114–21. 10.1093/rheumatology/kep155 19589891

[7] van der Helm-van Mil AH Detert J Le Cessie S Filer A Bastian H Burmester GR Validation of a prediction rule for disease outcome in patients with recent-onset undifferentiated arthritis: moving toward individualized treatment decision-making. Arthritis Rheum (2008) 58(8):2241–7. 10.1002/art.23681 18668546

[8] Kuriya B Cheng CK Chen HM Bykerk VP. Validation of a prediction rule for development of rheumatoid arthritis in patients with early undifferentiated arthritis. Ann Rheum Dis (2009) 68(9):1482–5. 10.1136/ard.2008.092676 19015211

[9] Aletaha D Neogi T Silman A Funovits J Felson D Bingham C 2010 rheumatoid arthritis classification criteria. Arthritis Rheum (2010) 62(9):2569–81. 10.1002/art.27584 20872595

[10] Alves C Luime JJ van Zeben D Huisman A-M Weel AE Barendregt PJ Diagnostic performance of the ACR/EULAR 2010 criteria for rheumatoid arthritis and two diagnostic algorithms in an early arthritis clinic (REACH). Ann Rheum Dis (2011) 70:1645–7. 10.1136/ard.2010.142299 21622769 PMC3147228

[11] Van Der Linden MPM Batstra MR Bakker-Jonges LE Detert J Bastian H Scherer HU Toward a data-driven evaluation of the 2010 American College of Rheumatology/European League against rheumatism criteria for rheumatoid arthritis: is it sensible to look at levels of rheumatoid factor? Arthritis Rheum (2011) 63(5):1190–9. 10.1002/art.30200 21538311

[12] Breedveld FC Emery P Keystone E Patel K Furst DE Kalden JR Infliximab in active early rheumatoid arthritis. Ann Rheum Dis (2004) 63:149–55. 10.1136/ard.2003.013961 14722203 PMC1754899

[13] Landewe RB Boers M Verhoeven AC Westhovens R van de Laar MA Markusse HM COBRA combination therapy in patients with early rheumatoid arthritis: long-term structural benefits of a brief intervention. Arthritis Rheum (2002) 46(2):347–56. 10.1002/art.10083 11840436

[14] Smolen JS Van Der Heijde DM St Clair EW Emery P Bathon JM Keystone E Predictors of joint damage in patients with early rheumatoid arthritis treated with high-dose methotrexate with or without concomitant infliximab: results from the ASPIRE trial. Arthritis Rheum (2006) 54(3):702–10. 10.1002/art.21678 16508926

[15] Landewé R van der Heijde D Klareskog L van Vollenhoven R Fatenejad S. Disconnect between inflammation and joint destruction after treatment with etanercept plus methotrexate: results from the trial of etanercept and methotrexate with radiographic and patient outcomes. Arthritis Rheum (2006) 54(10):3119–25. 10.1002/art.22143 17009230

[16] Smolen JS Han C Bala M Maini RN Kalden JR van der Heijde D Evidence of radiographic benefit of treatment with infliximab plus methotrexate in rheumatoid arthritis patients who had no clinical improvement: a detailed subanalysis of data from the anti-tumor necrosis factor trial in rheumatoid arthritis with concomitant therapy study. Arthritis Rheum (2005) 52(4):1020–30. 10.1002/art.20982 15818697

[17] De Groof A Ducreux J Humby F Nzeusseu Toukap A Badot V Pitzalis C Higher expression of TNFalpha-induced genes in the synovium of patients with early rheumatoid arthritis correlates with disease activity, and predicts absence of response to first line therapy. Arthritis Res Ther (2016) 18:19 10.1186/s13075-016-0919-z 26792343 PMC4719339

[18] Lindqvist E Eberhardt K Bendtzen K Heinegård D Saxne T. Prognostic laboratory markers of joint damage in rheumatoid arthritis. Ann Rheum Dis (2005) 64(2):196–201. 10.1136/ard.2003.019992 15458956 PMC1755350

[19] Syversen SW Gaarder PI Goll GL Ødegård S Haavardsholm EA Mowinckel P High anti-cyclic citrullinated peptide levels and an algorithm of four variables predict radiographic progression in patients with rheumatoid arthritis: results from a 10-year longitudinal study. Ann Rheum Dis (2008) 67(2):212–7. 10.1136/ard.2006.068247 17526555

[20] Durnez A Vanderschueren G Lateur L Westhovens R Verschueren P. Effectiveness of initial treatment allocation based on expert opinion for prevention of rapid radiographic progression in daily practice of an early RA cohort. Ann Rheum Dis (2011) 70(4):634–7. 10.1136/ard.2010.135319 21177296

[21] Visser K Goekoop-Ruiterman YP de Vries-Bouwstra JK Ronday HK Seys PE Kerstens PJ A matrix risk model for the prediction of rapid radiographic progression in patients with rheumatoid arthritis receiving different dynamic treatment strategies: post hoc analyses from the BeSt study. Ann Rheum Dis (2010) 69(7):1333–7. 10.1136/ard.2009.121160 20498212

[22] Lillegraven S Paynter N Prince FH Shadick NA Haavardsholm EA Frits ML Performance of matrix-based risk models for rapid radiographic progression in a cohort of patients with established rheumatoid arthritis. Arthritis Care Res (2013) 65(4):526–33. 10.1002/acr.21870 23044765 PMC3594116

[23] de Punder YM van Riel PL Fransen J. A simplified baseline prediction model for joint damage progression in rheumatoid arthritis: a step toward personalized medicine. J Rheumatol (2015) 42(3):391–7. 10.3899/jrheum.140327 25593237

[24] Hambardzumyan K Bolce R Saevarsdottir S Cruickshank SE Sasso EH Chernoff D Pretreatment multi-biomarker disease activity score and radiographic progression in early RA: results from the SWEFOT trial. Ann Rheum Dis (2015) 74(6):1102–9. 10.1136/annrheumdis-2013-204986 24812287 PMC4431327

[25] Ostendorf B Scherer A Mödder U Schneider M. Diagnostic value of magnetic resonance imaging of the forefeet in early rheumatoid arthritis when findings on imaging of the metacarpophalangeal joints of the hands remain normal. Arthritis Rheum (2004) 50(7):2094–102. 10.1002/art.20314 15248206

[26] Brown AK Quinn MA Karim Z Conaghan PG Peterfy CG Hensor E Presence of significant synovitis in rheumatoid arthritis patients with disease-modifying antirheumatic drug-induced clinical remission: evidence from an imaging study may explain structural progression. Arthritis Rheum (2006) 54(12):3761–73. 10.1002/art.22190 17133543

[27] McQueen FM Benton N Perry D Crabbe J Robinson E Yeoman S Bone edema scored on magnetic resonance imaging scans of the dominant carpus at presentation predicts radiographic joint damage of the hands and feet six years later in patients with rheumatoid arthritis. Arthritis Rheum (2003) 48(7):1814–27. 10.1002/art.11162 12847674

[28] Suter LG Fraenkel L Braithwaite RS. Role of magnetic resonance imaging in the diagnosis and prognosis of rheumatoid arthritis. Arthritis Care Res (Hoboken) (2011) 63(5):675–88. 10.1002/acr.20409 21557523 PMC3135707

[29] Hetland ML Ejbjerg B Hørslev-Petersen K Jacobsen S Vestergaard A Jurik AG MRI bone oedema is the strongest predictor of subsequent radiographic progression in early rheumatoid arthritis. Results from a 2-year randomised controlled trial (CIMESTRA). Ann Rheum Dis (2009) 68(3):384–90. 10.1136/ard.2008.088245 18388160

[30] Duer-Jensen A Hørslev-Petersen K Hetland ML Bak L Ejbjerg BJ Hansen MS Bone edema on magnetic resonance imaging is an independent predictor of rheumatoid arthritis development in patients with early undifferentiated arthritis. Arthritis Rheum (2011) 63(8):2192–202. 10.1002/art.30396 21484772

[31] Tamai M Kawakami A Uetani M Takao S Arima K Iwamoto N A prediction rule for disease outcome in patients with undifferentiated arthritis using magnetic resonance imaging of the wrists and finger joints and serologic autoantibodies. Arthritis Rheum (2009) 61(6):772–8. 10.1002/art.24711 19479686

[32] Narváez J Sirvent E Narváez JA Bas J Gómez-Vaquero C Reina D Usefulness of magnetic resonance imaging of the hand versus anticyclic citrullinated peptide antibody testing to confirm the diagnosis of clinically suspected early rheumatoid arthritis in the absence of rheumatoid factor and radiographic erosions. Semin Arthritis Rheum (2008) 38(2):101–9. 10.1016/j.semarthrit.2007.10.012 18221987

[33] Keen HI Brown AK Wakefield RJ Conaghan PG. MRI and musculoskeletal ultrasonography as diagnostic tools in early arthritis. Rheum Dis Clin North Am (2005) 31(4):699–714. 10.1016/j.rdc.2005.07.002 16287592

[34] Freeston JE Wakefield RJ Conaghan PG Hensor EM Stewart SP Emery P. A diagnostic algorithm for persistence of very early inflammatory arthritis: the utility of power Doppler ultrasound when added to conventional assessment tools. Ann Rheum Dis (2010) 69(2):417–9. 10.1136/ard.2008.106658 19359260

[35] Filer A de Pablo P Allen G Nightingale P Jordan A Jobanputra P Utility of ultrasound joint counts in the prediction of rheumatoid arthritis in patients with very early synovitis. Ann Rheum Dis (2011) 70(3):500–7. 10.1136/ard.2010.131573 21115552 PMC3033529

[36] Pratt AG Lorenzi AR Wilson G Platt PN Isaacs JD. Predicting persistent inflammatory arthritis amongst early arthritis clinic patients in the UK: is musculoskeletal ultrasound required? Arthritis Res Ther (2013) 15(5):R118. 10.1186/ar4298 24028567 PMC3978649

[37] Braun-Moscovici Y Markovits D Zinder O Schapira D Rozin A Ehrenburg M Anti-cyclic citrullinated protein antibodies as a predictor of response to anti-tumor necrosis factor-alpha therapy in patients with rheumatoid arthritis. J Rheumatol (2006) 33(3):497–500. 16511906

[38] Mancarella L Bobbio-Pallavicini F Ceccarelli F Falappone PC Ferrante A Malesci D Good clinical response, remission, and predictors of remission in rheumatoid arthritis patients treated with tumor necrosis factor-alpha blockers: the GISEA study. J Rheumatol (2007) 34(8):1670–3. 17611987

[39] Potter C Hyrich KL Tracey A Lunt M Plant D Symmons DP Association of rheumatoid factor and anti-cyclic citrullinated peptide positivity, but not carriage of shared epitope or PTPN22 susceptibility variants, with anti-tumour necrosis factor response in rheumatoid arthritis. Ann Rheum Dis (2009) 68(1):69–74. 10.1136/ard.2007.084715 18375541 PMC2596303

[40] Buch MH Smolen JS Betteridge N Breedveld FC Burmester G Dörner T Updated consensus statement on the use of rituximab in patients with rheumatoid arthritis. Ann Rheum Dis (2011) 70(6):909–20. 10.1136/ard.2010.144998 21378402 PMC3086093

[41] Lal P Su Z Holweg CT Silverman GJ Schwartzman S Kelman A Inflammation and autoantibody markers identify rheumatoid arthritis patients with enhanced clinical benefit following rituximab treatment. Arthritis Rheum (2011) 63(12):3681–91. 10.1002/art.30596 22127691

[42] Isaacs JD Cohen SB Emery P Tak PP Wang J Lei G Effect of baseline rheumatoid factor and anticitrullinated peptide antibody serotype on rituximab clinical response: a meta-analysis. Ann Rheum Dis (2013) 72(3):329–36. 10.1136/annrheumdis-2011-201117 22689315

[43] Gottenberg JE Courvoisier DS Hernandez MV Iannone F Lie E Canhão H Brief report: association of rheumatoid factor and anti-citrullinated protein antibody positivity with better effectiveness of abatacept: results from the Pan-European Registry Analysis. Arthritis Rheumatol (2016) 68:1346–52. 10.1002/art.39595 26815727

[44] Nüßlein HG Alten R Galeazzi M Lorenz HM Boumpas D Nurmohamed MT Real-world effectiveness of abatacept for rheumatoid arthritis treatment in European and Canadian populations: a 6-month interim analysis of the 2-year, observational, prospective ACTION study. BMC Musculoskelet Disord (2014) 15(1):14. 10.1186/1471-2474-15-14 24410774 PMC3898027

[45] Forsblad-d’Elia H Bengtsson K Kristensen LE Jacobsson LTH Drug adherence, response and predictors thereof for tocilizumab in patients with rheumatoid arthritis: results from the Swedish biologics register. Rheumatology (Oxford) (2015) 54(7):1186–93. 10.1093/rheumatology/keu455 25505001

[46] Varache S Cornec D Morvan J Devauchelle-Pensec V Berthelot J-M Le Henaff-Bourhis C Diagnostic accuracy of ACR/EULAR 2010 criteria for rheumatoid arthritis in a 2-Year Cohort. J Rheumatol (2011) 38(7):1250–7. 10.3899/jrheum.101227 21572146

[47] Stott DI Hiepe F Hummel M Steinhauser G Berek C Antigen-driven clonal proliferation of B cells within the target tissue of an autoimmune disease. The salivary glands of patients with Sjögren’s syndrome. J Clin Invest (1998) 102(5):938–46. 10.1172/JCI3234 9727062 PMC508959

[48] Serafini B Rosicarelli B Magliozzi R Stigliano E Aloisi F. Detection of ectopic B-cell follicles with germinal centers in the meninges of patients with secondary progressive multiple sclerosis. Brain Pathol (2004) 14(2):164–74. 10.1111/j.1750-3639.2004.tb00049.x 15193029 PMC8095922

[49] Marinkovic T Garin A Yokota Y Fu Y-X Ruddle NH Furtado GC Interaction of mature CD3+CD4+ T cells with dendritic cells triggers the development of tertiary lymphoid structures in the thyroid. J Clin Invest (2006) 116(10):2622–32. 10.1172/JCI28993 16998590 PMC1570377

[50] Randen I Mellbye OJ Forre O Natvig JB. The identification of germinal centres and follicular dendritic cell networks in rheumatoid synovial tissue. Scand J Immunol (1995) 41(5):481–6. 10.1111/j.1365-3083.1995.tb03596.x 7725067

[51] Humby F Bombardieri M Manzo A Kelly S Blades MC Kirkham B Ectopic lymphoid structures support ongoing production of class-switched autoantibodies in rheumatoid synovium. PLoS Med (2009) 6(1):e1. 10.1371/journal.pmed.0060001 19143467 PMC2621263

[52] Muramatsu M Kinoshita K Fagarasan S Yamada S Shinkai Y Honjo T Class switch recombination and hypermutation require activation-induced cytidine deaminase (AID), a potential RNA editing enzyme. Cell (2000) 102(5):553–63. 10.1016/S0092-8674(00)00078-7 11007474

[53] Kotake S Udagawa N Hakoda M Mogi M Yano K Tsuda E Activated human T cells directly induce osteoclastogenesis from human monocytes: possible role of T cells in bone destruction in rheumatoid arthritis patients. Arthritis Rheum (2001) 44(5):1003–12. 10.1002/1529-0131(200105)44:5<1003::AID-ANR179>3.3.CO;2-R 11352231

[54] Klimiuk PA Sierakowski S Latosiewicz R Skowronski J Cylwik JP Cylwik B Histological patterns of synovitis and serum chemokines in patients with rheumatoid arthritis. J Rheumatol (2005) 32(9):1666–72. 16142858

[55] Kraan MC Haringman JJ Post WJ Versendaal J Breedveld FC Tak PP. Immunohistological analysis of synovial tissue for differential diagnosis in early arthritis. Rheumatology (Oxford) (1999) 38(11):1074–80. 10.1093/rheumatology/38.11.1074 10556258

[56] Thurlings RM Wijbrandts CA Mebius RE Cantaert T Dinant HJ van der Pouw-Kraan TC Synovial lymphoid neogenesis does not define a specific clinical rheumatoid arthritis phenotype. Arthritis Rheum (2008) 58(6):1582–9. 10.1002/art.23505 18512774

[57] Cañete JD Celis R Moll C Izquierdo E Marsal S Sanmartí R Clinical significance of synovial lymphoid neogenesis and its reversal after anti-tumour necrosis factor α therapy in rheumatoid arthritis. Ann Rheum Dis (2009) 68(5):751–6. 10.1136/ard.2008.089284 18495732

[58] van de Sande MG Thurlings RM Boumans MJ Wijbrandts CA Modesti MG Gerlag DM Presence of lymphocyte aggregates in the synovium of patients with early arthritis in relationship to diagnosis and outcome: is it a constant feature over time? Ann Rheum Dis (2011) 70(4):700–3. 10.1136/ard.2010.139287 21173012

[59] Edwards JCW Leandro MJ Lymphoid follicles in joints: what do they mean? Arthritis Rheum (2008) 58(6):1563–5. 10.1002/art.23506 18512771

[60] Linn-Rasker SP van der Helm-van Mil AHM Breedveld FC Huizinga TWJ Arthritis of the large joints – in particular, the knee – at first presentation is predictive for a high level of radiological destruction of the small joints in rheumatoid arthritis. Ann Rheum Dis (2007) 66(5):646–50. 10.1136/ard.2006.066704 17142384 PMC1954616

[61] Rosengren S Wei N Kalunian KC Zvaifler NJ Kavanaugh A Boyle DL. Elevated autoantibody content in rheumatoid arthritis synovia with lymphoid aggregates and the effect of rituximab. Arthritis Res Ther (2008) 10(5):R105. 10.1186/ar2497 18761748 PMC2592782

[62] Pitzalis C Jones GW Bombardieri M Jones SA. Ectopic lymphoid-like structures in infection, cancer and autoimmunity. Nat Rev Immunol (2014) 14(7):447–62. 10.1038/nri3700 24948366

[63] Koczan D Drynda S Hecker M Drynda A Guthke R Kekow J Molecular discrimination of responders and nonresponders to anti-TNFalpha therapy in rheumatoid arthritis by etanercept. Arthritis Res Ther (2008) 10(3):R50–50. 10.1186/ar2419 18454843 PMC2483439

[64] Lequerré T Gauthier-Jauneau AC Bansard C Derambure C Hiron M Vittecoq O Gene profiling in white blood cells predicts infliximab responsiveness in rheumatoid arthritis. Arthritis Res Ther (2006) 8(4):R105–105. 10.1186/ar1990 16817978 PMC1779405

[65] Sekiguchi N Kawauchi S Furuya T Inaba N Matsuda K Ando S Messenger ribonucleic acid expression profile in peripheral blood cells from RA patients following treatment with an anti-TNF-α monoclonal antibody, infliximab. Rheumatology (Oxford) (2008) 47(6):780–8. 10.1093/rheumatology/ken083 18388148

[66] Marotte H Maslinski W Miossec P Circulating tumour necrosis factor-$α$ bioactivity in rheumatoid arthritis patients treated with infliximab: link to clinical response. Arthritis Res Ther (2004) 7(1):R149 10.1186/ar1465 15642135 PMC1064892

[67] van Baarsen LG Wijbrandts CA Gerlag DM Rustenburg F van der Pouw Kraan TC Dijkmans BA Pharmacogenomics of infliximab treatment using peripheral blood cells of patients with rheumatoid arthritis. Genes Immun (2010) 11(8):622–9. 10.1038/gene.2010.34 20555356

[68] Raterman HG Vosslamber S de Ridder S Nurmohamed MT Lems WF Boers M The interferon type I signature towards prediction of non-response to rituximab in rheumatoid arthritis patients. Arthritis Res Ther (2012) 14(2):R95. 10.1186/ar3819 22540992 PMC3446469

[69] Matsumoto M Fu Y-X Molina H Huang G Kim J Thomas DA Distinct roles of lymphotoxin α and the type I tumor necrosis factor (TNF) receptor in the establishment of follicular dendritic cells from non-bone marrow-derived cells. J Exp Med (1997) 186(12):1997L–2004. 10.1084/jem.186.12.1997 9396768 PMC2199170

[70] Klaasen R Thurlings RM Wijbrandts CA van Kuijk AW Baeten D Gerlag DM The relationship between synovial lymphocyte aggregates and the clinical response to infliximab in rheumatoid arthritis: a prospective study. Arthritis Rheum (2009) 60(11):3217–24. 10.1002/art.24913 19877042

[71] Licastro F Chiappelli M Ianni M Porcellini E. Tumor necrosis factor-alpha antagonists: differential clinical effects by different biotechnological molecules. Int J Immunopathol Pharmacol (2009) 22:567–72. 10.1177/039463200902200302 19822073

[72] Ulfgren AK Andersson U Engström M Klareskog L Maini RN Taylor PC. Systemic anti-tumor necrosis factor alpha therapy in rheumatoid arthritis down-regulates synovial tumor necrosis factor alpha synthesis. Arthritis Rheum (2000) 43(11):2391–6. 10.1002/1529-0131(200011)43:11<2391::AID-ANR3>3.0.CO;2-F 11083259

[73] Wijbrandts CA Dijkgraaf MGW Kraan MC Vinkenoog M Smeets TJ Dinant H The clinical response to infliximab in rheumatoid arthritis is in part dependent on pretreatment tumour necrosis factor α expression in the synovium. Ann Rheum Dis (2008) 67(8):1139–44. 10.1136/ard.2007.080440 18055470 PMC2564801

[74] Lindberg J af Klint E Catrina AI Nilsson P Klareskog L Ulfgren AK Effect of infliximab on mRNA expression profiles in synovial tissue of rheumatoid arthritis patients. Arthritis Res Ther (2006) 8(6):R179. 10.1186/ar1903 17134501 PMC1794525

[75] van der Pouw Kraan TC Wijbrandts CA van Baarsen LG Rustenburg F Baggen JM Verweij CL Responsiveness to anti-tumour necrosis factor α therapy is related to pre-treatment tissue inflammation levels in rheumatoid arthritis patients. Ann Rheum Dis (2008) 67(4):563–6. 10.1136/ard.2007.081950 18042642

[76] Badot V Galant C Nzeusseu Toukap A Theate I Maudoux AL den Eynde BJ Gene expression profiling in the synovium identifies a predictive signature of absence of response to adalimumab therapy in rheumatoid arthritis. Arthritis Res Ther (2009) 11(2):R57. 10.1186/ar2678 19389237 PMC2688209

[77] Lindberg J Wijbrandts CA van Baarsen LG Nader G Klareskog L Catrina A The gene expression profile in the synovium as a predictor of the clinical response to infliximab treatment in rheumatoid arthritis. PLoS One (2010) 5(6):e11310. 10.1371/journal.pone.0011310 20593016 PMC2892481

[78] Dennis G Holweg CT Kummerfeld SK Choy DF Setiadi AF Hackney JA Synovial phenotypes in rheumatoid arthritis correlate with response to biologic therapeutics. Arthritis Res Ther (2014) 16(2):R90. 10.1186/ar4555 25167216 PMC4060385

[79] Pitzalis C Kelly S Humby F. New learnings on the pathophysiology of RA from synovial biopsies. Curr Opin Rheumatol (2013) 25(3):334–44. 10.1097/BOR.0b013e32835fd8eb 23492740

[80] Edwards JC Szczepanski L Szechinski J Filipowicz-Sosnowska A Emery P Close DR Efficacy of B-cell-targeted therapy with rituximab in patients with rheumatoid arthritis. N Engl J Med (2004) 350(25):2572–81. 10.1056/NEJMoa032534 15201414

[81] Cohen SB Keystone E Genovese MC Emery P Peterfy C Tak PP Continued inhibition of structural damage over 2 years in patients with rheumatoid arthritis treated with rituximab in combination with methotrexate. Ann Rheum Dis (2010) 69(6):1158–61. 10.1136/ard.2009.119222 20439295 PMC2935326

[82] Emery P Fleischmann R Filipowicz-Sosnowska A Schechtman J Szczepanski L Kavanaugh A The efficacy and safety of rituximab in patients with active rheumatoid arthritis despite methotrexate treatment: results of a phase IIB randomized, double-blind, placebo-controlled, dose-ranging trial. Arthritis Rheum (2006) 54(5):1390–400. 10.1002/art.21778 16649186

[83] Mease PJ Cohen S Gaylis NB Chubick A Kaell AT Greenwald M Efficacy and safety of retreatment in patients with rheumatoid arthritis with previous inadequate response to tumor necrosis factor inhibitors: results from the SUNRISE trial. J Rheumatol (2010) 37(5):917–27. 10.3899/jrheum.090442 20194448

[84] Kavanaugh A Rosengren S Lee SJ Hammaker D Firestein GS Kalunian K Assessment of rituximab’s immunomodulatory synovial effects (ARISE trial). 1: clinical and synovial biomarker results. Ann Rheum Dis (2008) 67(3):402–8. 10.1136/ard.2007.074229 17644541 PMC2754142

[85] Thurlings RM Vos K Wijbrandts CA Zwinderman AH Gerlag DM Tak PP. Synovial tissue response to rituximab: mechanism of action and identification of biomarkers of response. Ann Rheum Dis (2008) 67(7):917–25. 10.1136/ard.2007.080960 17965121 PMC2564787

[86] Dass S Rawstron AC Vital EM Henshaw K McGonagle D Emery P. Highly sensitive B cell analysis predicts response to rituximab therapy in rheumatoid arthritis. Arthritis Rheum (2008) 58(10):2993–9. 10.1002/art.23902 18821683

[87] Buch MH Boyle DL Rosengren S Saleem B Reece RJ Rhodes LA Mode of action of abatacept in rheumatoid arthritis patients having failed tumour necrosis factor blockade: a histological, gene expression and dynamic magnetic resonance imaging pilot study. Ann Rheum Dis (2009) 68(7):1220–7. 10.1136/ard.2008.091876 18772191 PMC2689522

[88] Kaufmann M Pusztai L Biedenkopf Expert Panel Members. Use of standard markers and incorporation of molecular markers into breast cancer therapy: consensus recommendations from an international expert panel. Cancer (2011) 117(8):1575–82. 10.1002/cncr.25660 21472705

[89] Gangadhar T Schilsky RL. Molecular markers to individualize adjuvant therapy for colon cancer. Nat Rev Clin Oncol (2010) 7(6):318–25. 10.1038/nrclinonc.2010.62 20440283

